# Climate Vulnerability Index and Incident Type 2 Diabetes in a Large Integrated Health Care System

**DOI:** 10.1001/jamanetworkopen.2025.47119

**Published:** 2025-12-05

**Authors:** Jad Ardakani, Izza Shahid, Rakesh Gullapelli, Lindsey Russo, Budhaditya Bose, Juan C. Nicolas, Zulqarnain Javed, Weichuan Dong, Jay E. Maddock, Arnab K. Ghosh, Grace Tee Lewis, Stephen Jones, Archana Sadhu, Sanjay Rajagopalan, Khurram Nasir, Sadeer Al-Kindi

**Affiliations:** 1 Center for Cardiovascular Computational and Precision Health (C3PH), Department of Cardiology, Houston Methodist DeBakey Heart & Vascular Center, Houston, Texas; 2 Department of Medicine, Weill Cornell Medicine, New York, New York; 3 Center for Health Data Science & Analytics, Houston Methodist, Houston, Texas; 4 Center for Health & Nature, Houston, Texas; 5 Environmental Defense Fund, Houston, Texas; 6 Department of Medicine, Houston Methodist Hospital, Houston, Texas; 7 Harrington Heart and Vascular Institute, University Hospitals, Cleveland, Ohio; 8 Case Cardiovascular Research Institute, Case Western Reserve University, Cleveland, Ohio; 9 Methodist-Rice Digital Health Institute, Houston Methodist, Houston, Texas

## Abstract

**Importance:**

Place-based Climate Vulnerability Index (CVI) may shape metabolic risk through environmental and socioeconomic stressors, but its association with incident type 2 diabetes (T2D) is not well characterized.

**Objective:**

To determine whether residence in communities with higher CVI is associated with risk of incident T2D.

**Design, Setting, and Participants:**

This retrospective cohort study used data from the Houston Methodist Learning Health System Registry, a large integrated health system that primarily serves Greater Houston, Texas. Participants included adults aged 18 years or older without T2D at baseline and with at least 1 outpatient encounter and at least 1 subsequent health care encounter from June 2016 to August 2023. Data analysis was conducted September 2025.

**Exposures:**

US census tract–level CVI categorized into quartiles (Q1-Q4).

**Main Outcomes and Measures:**

The primary outcome was incident T2D identified by *International Statistical Classification of Diseases, Tenth Revision, Clinical Modification *codes, antihyperglycemic prescriptions, or hemoglobin A_1c_ 6.5% or higher. Primary measures were incidence rates (cases per 100 person-years) and adjusted hazard ratios (HRs) with 95% CIs comparing Q4 vs Q1 from Cox models adjusted for demographics, insurance, cardiometabolic risk factors, and baseline hemoglobin A_1c_.

**Results:**

Among 1 003 526 participants (mean [SD] age, 50.9 [18.4] years; 605 829 women [60.4%]; 132 451 African American or Black [13.2%]; 71 408 Asian [7.1%]; 156 989 Hispanic or Latinx [15.6%]; 566 632 White [56.5%]; 35 565 other [3.5%]; 42 942 unknown or not reported [4.3%]), 40 152 developed T2D over 2.1 million person-years (overall incidence, 1.88 cases per 100 person-years). Diabetes incidence was higher among participants residing in Q4 vs Q1 CVI areas (2.66 vs 1.48 cases per 100 person-years), and the 7-year risk was 14.1% for Q4 participants vs 8.6% for Q1 participants. Residence in Q4 vs Q1 CVI was associated with higher T2D risk (HR, 1.23; 95% CI, 1.11-1.36), a statistically significant difference.

**Conclusions and Relevance:**

In this cohort study of 1 003 526 adults, higher community CVI was associated with greater risk of incident T2D independently of traditional risk factors. Linking geocoded CVI to electronic health records may support targeted prevention, risk stratification, and population health planning.

## Key Points

**Question:**

Is residence in communities with a higher Climate Vulnerability Index (CVI) associated with an increased risk of incident type 2 diabetes?

**Findings:**

In this retrospective cohort study of more than 1 million adults without baseline diabetes, residence in the highest vs lowest CVI quartile was associated with higher diabetes incidence (2.66 vs 1.48 cases per 100 person-years; 14.1% vs 8.6% at 7 years), a statistically significant difference.

**Meaning:**

These findings suggest that living in higher-CVI communities is associated with greater diabetes risk; incorporating place-based vulnerability into clinical risk stratification and public health planning may help target prevention.

## Teaser

This cohort study examines whether residence in a community with a high Climate Vulnerability Index is associated with risk of incident type 2 diabetes.

## Introduction

Type 2 diabetes (T2D) and obesity remain among the most important global public health challenges, driving substantial morbidity, mortality, and health care cost.^1^ Although conventional risk factors for diabetes, such as sedentary lifestyle and poor diet, are well recognized,^2^ there is growing interest in understanding broader social and environmental determinants influencing T2D risk and outcomes.^3,4,5,6^ Social determinants of health (SDOH), including economic status, education, and community infrastructure, have increasingly been recognized as contributors to chronic metabolic disorders, including T2D.^7^

Environmental exposures have also been increasingly recognized as contributors to T2D risk.^6,8^ Prior studies associated T2D risk with air pollution,^9,10^ noise pollution,^11^ light exposure,^12^ metals,^13^ proximity to hazardous waste sites,^14^ extreme heat,^15^ and historical redlining.^16^ A Lancet Commission report described the epidemics of obesity, malnutrition, and climate change as a *syndemic*, highlighting their shared origins and overlapping social and biological drivers.^17^

The Climate Vulnerability Index (CVI)^18^ offers a comprehensive measure of community susceptibility to climate-driven risks (extreme weather and long-term ecological shifts) and baseline vulnerabilities (socioeconomic conditions, infrastructure resilience, and health status). By integrating 184 distinct indicators at the US census tract level, the CVI quantifies vulnerability across 7 domains: baseline health, socioeconomic, infrastructure, environment, and climate health, socioeconomic, and extreme.^18,19^

Recent work has demonstrated associations between CVI and cardiometabolic outcomes, including cardiovascular, kidney, and metabolic disease, supporting the importance of exploring its relationship with diabetes.^20^ In contrast, most existing studies focus on acute and immediate health outcomes associated with climate-amplified events, such as heat-related illnesses and respiratory conditions.^21^ Texas, a US state marked by extreme heat, flooding, wildfires, and pronounced socioeconomic disparities, provides an ideal setting for this investigation.^22^ In this study, we examine the association between CVI and incident T2D in a large, diverse cohort within an integrated health care system, aiming to clarify how cumulative climate and socioeconomic vulnerabilities may impact T2D risk.

## Methods

### Study Design and Data Source

This retrospective cohort study used data from the Houston Methodist Cardiovascular Disease Learning Health System (CVD-LHS) Registry. Adults aged 18 years or older with at least 1 outpatient encounter and 1 subsequent health care encounter between June 2016 and August 2023 were included, with up to 7 years of follow-up. The CVD-LHS Registry contains deidentified clinical, laboratory, imaging, and pharmacy data for patients receiving care at Houston Methodist Hospitals and clinics, which primarily serve Greater Houston, Texas, with a minority of patients residing elsewhere in Texas or out of state.^23^ Registry design and methods have been detailed previously.^23^ This study was approved by the institutional review board at Houston Methodist with a waiver of informed consent and followed Strengthening the Reporting of Observational Studies in Epidemiology (STROBE) reporting guidelines for cohort studies.

### Study Population

The study included all adult patients (aged ≥18 years) with at least 1 outpatient encounter and 1 subsequent health care encounter during the study period. Patients with baseline T2D, defined by a combination of *International Statistical Classification of Diseases, Tenth Revision, Clinical Modification* codes and prescriptions for diabetes-related medications or hemoglobin A_1c_ (HbA_1c_) 6.5% or higher (to convert HbA_1c_ to proportion of total hemoglobin, multiply by 0.01), before or at their first encounter, were excluded (eAppendix and eFigure 1 in Supplement 1). Codes were documented in visit diagnoses, problem lists, or comorbidity lists, and incident T2D was defined as the first new occurrence after baseline during follow-up.

Residential addresses were geocoded in ArcGIS using the most recent home address recorded in the electronic health record. US Census Bureau TIGER/Line shapefiles were used to assign each patient an 11-digit Federal Information Processing Series census tract code, which was then linked to the corresponding CVI score for that census tract. Tract-level CVI variability and patient density are displayed in eFigure 2 in Supplement 1.

### Demographic and Clinical Characteristics

Sex and race and ethnicity were self-reported during routine administrative intake and recorded as discrete fields in the electronic health record. Categories were listed alphabetically as African American or Black, Asian, Hispanic or Latinx, White, other, and unknown or not reported. The CVD-LHS Registry’s other category included Hawaiian or Pacific Islander, Native American, null (ie, the field was missing), and other. Race and ethnicity were included to describe the cohort and adjust for potential confounding related to social and structural determinants of health.

### Climate Vulnerability Index

The CVI^18^ is a comprehensive, data-driven composite measure that quantifies community-level vulnerability to climate-related risks across the US. Developed by Tee Lewis et al^18^ through a multidisciplinary effort involving environmental health, climate science, and socioeconomic research, the CVI integrates 184 indicators from national datasets, including the Centers for Disease Control and Prevention, Environmental Protection Agency, US Census Bureau, Federal Emergency Management Agency, and additional federal and local databases.

The index comprises 7 domains: 4 baseline vulnerability domains (socioeconomic status, household composition and disability, minority status and language, and housing type and transportation) and 3 climate-specific vulnerability domains (climate extremes, environmental exposures, and infrastructure resilience).^18,19^ These domains capture vulnerability to both acute hazards (hurricanes, flooding, and heatwaves) and chronic SDOH (poverty, unemployment, linguistic isolation, limited access to health care and healthy food sources, and proximity to pollutants).

Each indicator was assigned at the US census tract level, enabling high spatial resolution for linking community conditions to health outcomes. Indicators were standardized, weighted, and aggregated into a composite score ranging from 0 to 1, with higher values indicating greater vulnerability. For this analysis, CVI scores were stratified into quartiles (Q1-Q4) according to the distribution of scores within the study cohort, with Q4 representing the most vulnerable communities. A full description of CVI development and indicator selection is available in Tee Lewis et al.^18^

### Statistical Analysis

Baseline demographic and clinical characteristics were compared across CVI quartiles. Continuous variables were summarized as mean (SD) and compared across using 1-way analysis of variance, whereas categorical variables were summarized as counts (percentages) and compared using χ^2^ tests.

Kaplan-Meier curves were constructed to estimate cumulative T2D incidence by CVI quartile, with group differences assessed by the log-rank test. Patients were censored at their last follow-up or death.

Cox proportional hazards models were subsequently used to evaluate the association between CVI quartiles and incident T2D. Four models were sequentially constructed: model 1, unadjusted; model 2, adjusted for age, sex, and race and ethnicity; model 3, additionally adjusted for insurance and cardiovascular risk factors (hypertension, obesity, dyslipidemia, and smoking); and model 4, further adjusted for baseline HbA_1c_. Models 1 to 3 included the full analytic cohort. Forest plots displayed hazard ratios (HRs) and 95% CIs for each model (eFigure 3 in Supplement 1). Body mass index (BMI; calculated as weight in kilograms divided by height in meters squared and stratified as normal [18.5 to <25.0], overweight [25.0 to <30.0], and obesity [≥30.0]), blood pressure, lipids, and HbA_1c_ were measured at baseline. Results were reported as HRs (95% CIs), with estimates rounded to 2 decimal places and *P* values are reported exactly (2 digits, or 3 if <.01). A 2-sided α = .05 was used. Patients with missing exposure, outcome, or covariate data were excluded.

Data cleaning involved removing implausible or placeholder values. Analyses were performed in R statistical software version 4.3.2 (R Project for Statistical Computing), using the survival, dplyr, tidyr, forestplot, grid, survminer, purrr, and broom packages. Data analysis was conducted in September 2025.

## Results

### Baseline Characteristics

Among 1 003 526 adults (mean [SD] age, 50.9 [18.4] years), 605 829 (60.4%) were women, 132 451 (13.2%) were African American or Black, 71 408 (7.1%) were Asian, 156 989 (15.6%) were Hispanic or Latinx, 566 632 (56.5%) were White, 35 565 (3.5%) were other races, and 42 942 (4.3%) were of unknown race or race was not reported. Baseline characteristics are summarized in Table 1.

**Table 1.** Baseline Characteristics of Study Population Across Climate Vulnerability Index Quartiles

Characteristics	Participants, No. (%)					*P* value
	Total (N = 1 003 526)	Quartile 1 (n = 250 882)	Quartile 2 (n = 250 882)	Quartile 3 (n = 250 881)	Quartile 4 (n = 250 881)	
Age, mean (SD), y	50.9 (18.4)	51.6 (18.2)	50.3 (18.3)	50.5 (18.5)	51.3 (18.6)	<.001
Sex						
Female	605 829 (60.4)	147 254 (58.7)	151 968 (60.6)	153 058 (61.0)	153 549 (61.2)	<.001
Male	397 668 (39.6)	103 618 (41.3)	98 911 (39.4)	97 812 (39.0)	97 327 (38.8)	<.001
Race and ethnicity						
African American or Black	132 451 (13.2)	21 620 (8.6)	25 343 (10.1)	35 715 (14.2)	49 773 (19.8)	<.001
Asian	71 408 (7.1)	30 748 (12.3)	17 293 (6.9)	15 106 (6.0)	8261 (3.3)	<.001
Hispanic or Latinx	156 989 (15.6)	25 328 (10.1)	31 036 (12.4)	40 908 (16.3)	58 717 (23.4)	<.001
White	566 632 (56.5)	153 774 (61.3)	156 347 (62.3)	138 784 (55.3)	117 727 (46.9)	<.001
Other^a^	35 565 (3.5)	9511 (3.8)	9775 (3.9)	9131 (3.6)	7148 (2.8)	<.001
Unknown or not reported	42 942 (4.3)	9901 (3.9)	11 088 (4.4)	11 237 (4.5)	9255 (3.7)	<.001
Ever smoked	151 623 (15.1)	32 615 (13.0)	34 083 (13.6)	38 478 (15.3)	46 447 (18.5)	<.001
Body mass index, mean (SD)^b^	28.68 (6.75)	27.57 (6.1)	28.06 (6.4)	29.03 (6.84)	30.07 (7.33)	<.001
Insurance						
Public	264 774 (26.4)	64 112 (25.6)	70 958 (28.3)	68 040 (27.1)	61 664 (24.6)	<.001
Private	434 148 (43.3)	113 499 (45.2)	110 287 (44.0)	107 351 (42.8)	103 011 (41.1)	<.001
Uninsured or other or not applicable	234 726 (23.4)	56 189 (22.4)	53 401 (21.3)	57 650 (23.0)	67 486 (26.9)	<.001
Self-pay	69 878 (7.0)	17 082 (6.8)	16 236 (6.5)	17 840 (7.1)	18 720 (7.5)	<.001
Hypertension	389 253 (38.8)	91 515 (36.5)	89 369 (35.6)	97 515 (38.9)	110 854 (44.2)	<.001
Dyslipidemia	297 490 (29.6)	79 859 (31.8)	75 933 (30.3)	73 207 (29.2)	68 491 (27.3)	<.001
Obesity	352 429 (35.1)	70 643 (28.2)	78 485 (31.3)	93 481 (37.3)	109 820 (43.8)	<.001
Hemoglobin A_1c_, mean (SD), % (n = 39 665)	5.46 (0.42)	5.45 (0.41)	5.44 (0.42)	5.46 (0.42)	5.49 (0.44)	<.001
Low-density lipoprotein, mean (SD), mg/dL (n = 443 787)	109.9 (35.9)	110.8 (35.6)	110.4 (35.5)	110.2 (35.8)	108.1 (36.6)	.06
High-density lipoprotein, mean (SD), mg/dL (n = 444 309)	56.5 (17.3)	57.9 (17.5)	57.8 (17.5)	55.9 (16.9)	54 (16.8)	<.001
First triglycerides, mean (SD), mg/dL (n = 440 176)	120.2 (92.3)	116.6 (84.9)	118.5 (89.8)	121.7 (95.2)	124.6 (99.7)	.06

SI conversion factors: To convert hemoglobin A_1c_ to proportion of total hemoglobin, multiply by 0.01; high-density lipoprotein to millimoles per liter, multiply by 0.0259; low-density lipoprotein to millimoles per liter, multiply by 0.0259; triglycerides to millimoles per liter, multiply by 0.0113.

^a^ Refers to Hawaiian or Pacific Islander, Native American, null (ie, the field was missing), and other.

^b^ Body mass index is calculated as weight in kilograms divided by height in meters squared (stratified as normal [18.5 to <25.0], overweight [25.0 to <30.0], and obesity [≥30.0]).

CVI scores ranged from 0.25 to 0.75 (median [IQR], 0.52 [0.47-0.57]). Cutoff points were 0.00 to 0.47 for Q1, 0.47 to 0.52 for Q1, 0.52 to 0.57 for Q3, and 0.57 to 1.00 for Q4. Participants were evenly distributed across quartiles (approximately 250 882 each).

Compared with Q1, those in Q4 were more likely to be women (153 549 participants [61.2%] vs 147 254 participants [58.7%]), Hispanic or Latinx (58 717 participants [23.4%] vs 25 328 participants [10.1%]), or African American or Black (49 773 participants [19.8%] vs 21 620 participants [8.6%]), and less likely to be White (117 727 participants [46.9%] vs 153 774 participants [61.3%]). They also had higher prevalence of hypertension (110 854 participants [44.2%] vs 91 515 participants [36.5%]), obesity (109 820 participants [43.8%] vs 70 643 participants [28.2%]), and smoking history (46 447 participants [18.6%] vs 32 615 participants [13.0%]), but lower dyslipidemia (68 491 participants [27.3%] vs 79 859 participants [31.8%]). All differences were statistically significant.

### Incidence Rates of T2D

**Figure 1.**
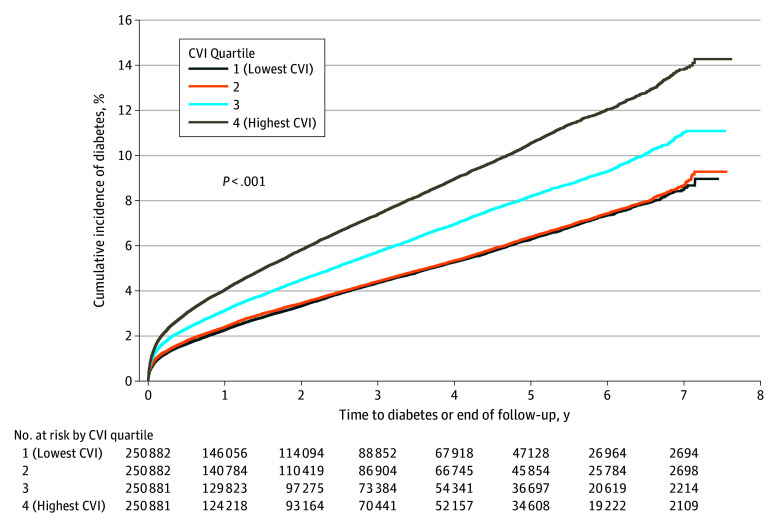
Cumulative Diabetes Incidence by Climate Vulnerability Index (CVI) Quartile

During the follow-up period of over 2.1 million person-years, 40 152 of 1 003 526 participants developed T2D, corresponding to an overall incidence rate of 1.88 cases per 100 person-years (95% CI, 1.87-1.90 cases per 100 person-years). A graded increase in T2D incidence was observed across CVI quartiles, from 1.48 cases per 100 person-years (95% CI, 1.44-1.51 cases per 100 person-years) in Q1 to 2.66 cases per 100 person-years (95% CI, 2.62-2.71 cases per 100 person-years) in Q4 (eTable 1 in Supplement 1). Kaplan-Meier analysis showed significantly higher cumulative incidence those in Q4 vs Q1 (Figure 1). By 7 years, 14.1% of participants in Q4 and 8.6% in Q1 had developed T2D.

Subgroup analyses (eTable 2 in Supplement 1) showed consistently higher incidence rates in men compared with women across all quartiles, particularly in Q4 (3.46 cases per 100 person-years [95% CI, 3.30-3.63 cases per 100 person-years] vs 2.24 cases per 100 person-years [95% CI, 2.12-2.36 cases per 100 person-years]). Incidence rates were also substantially higher among older adults (2.43 cases per 100 person-years [95% CI, 2.41-2.46 cases per 100 person-years] for those aged ≥50 years vs 1.03 cases per 100 person-years [95% CI, 1.01-1.05 cases per 100 person-years] for those aged <50 years) (eTable 2 in Supplement 1), and increased across the CVI quartiles within strata of HbA_1c_ and BMI (eFigure 4 in Supplement 1).

### Cox Models for Incident T2D

In unadjusted Cox regression models, individuals in Q4 had higher risk of developing T2D than those in Q1 (HR, 1.73; 95% CI, 1.68-1.78) (Table 2). The association remained after adjustment for age, sex, and race and ethnicity (HR, 1.67; 95% CI, 1.63-1.72) and persisted after further adjustment for insurance and cardiovascular risk factors (HR, 1.33; 95% CI, 1.29-1.37). In the fully adjusted model, which included baseline HbA_1c_ and comprised only 39 665 individuals, the HR was 1.23 (95% CI, 1.11-1.36) (Table 2; eFigure 3 in Supplement 1). When modeled continuously, each 0.10-unit (10%) increase in CVI was associated with a 41% higher risk of incident T2D (HR, 1.41; 95% CI, 1.39-1.43), supporting a graded association between increasing community vulnerability and diabetes risk.

**Table 2.** Cox Proportional Hazards Models for Climate Vulnerability Index Quartiles and Incident Type 2 Diabetes^a^

Model	Quartile 2		Quartile 3		Quartile 4	
	HR (95% CI)	*P* value	HR (95% CI)	*P* value	HR (95% CI)	*P* value
1	1.03 (1.00-1.06)	.09	1.33 (1.29-1.37)	<.001	1.73 (1.68-1.78)	<.001
2	1.06 (1.03-1.09)	<.001	1.34 (1.31-1.38)	<.001	1.67 (1.63-1.72)	<.001
3	1.02 (0.99-1.05)	.24	1.18 (1.15-1.21)	<.001	1.33 (1.29-1.37)	<.001
4	0.98 (0.88-1.09)	.72	1.14 (1.03-1.27)	.01	1.23 (1.11-1.36)	<.001

Abbreviation: HR, hazard ratio.

^a^ HRs compare quartiles 2 through 4 with quartile 1 (reference). Model 1 is unadjusted (n = 1 003 526); model 2 is adjusted for age, sex, race, and ethnicity (n = 1 003 526); model 3 is adjusted for insurance, hypertension, obesity, dyslipidemia, and smoking (n = 1 003 526); and model 4 is adjusted for baseline hemoglobin A_1c_ (n = 39 665).

### Subgroup Analyses

**Figure 2.**
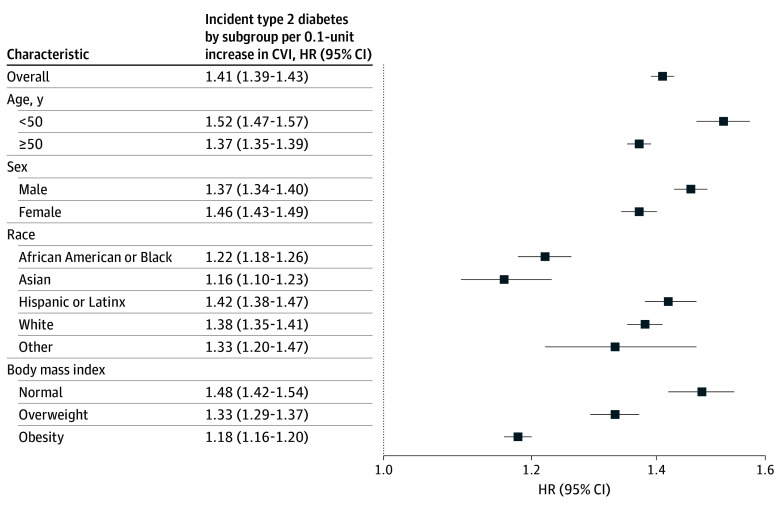
Hazard of Incident Diabetes by Subgroup Hazard ratios (HRs) and 95% CIs are shown per 0.1-unit increase in climate vulnerability index (CVI), modeled continuously. Referent is the baseline CVI (no increment). Estimates are stratified by age, sex, race, ethnicity, and body mass index (calculated as weight in kilograms divided by height in meters squared and stratified as normal [18.5 to <25.0], overweight [25.0 to <30.0], and obesity [≥30.0]). The dashed line indicates the null (HR = 1.00).

Associations between continuous CVI with T2D risk are shown in Figure 2. The magnitude of association varied across demographic and clinical subgroups. Point estimates were higher among individuals younger than 50 years (HR, 1.52; 95% CI, 1.47-1.57) than those aged 50 years and older (HR, 1.37; 95% CI, 1.35-1.39). Women exhibited a higher HR (HR, 1.46; 95% CI, 1.43-1.49) than men (HR, 1.37; 95% CI, 1.34-1.40). By race and ethnicity, estimates were higher among Hispanic or Latinx (HR, 1.42; 95% CI, 1.38-1.47) participants, White (HR, 1.38; 95% CI, 1.35-1.41) participants, and participants of other races (HR, 1.33; 95% CI, 1.20-1.47) and lower among African American or Black (HR, 1.22; 95% CI, 1.18-1.26) and Asian (HR, 1.16; 95% CI, 1.10-1.23) participants. When stratified by BMI, the HR was highest among individuals with normal BMI (HR, 1.48; 95% CI, 1.42-1.54), followed by those who were overweight (HR, 1.33; 95% CI, 1.29-1.37), and lowest among those with obesity (HR, 1.18; 95% CI, 1.16-1.20; *P* for interaction <.001).

### CVI Domain Analyses

Domain-specific associations were evaluated using model 3. As shown in eTable 3 in Supplement 1, baseline infrastructure had the highest HR for T2D risk (HR, 2.97; 95% CI, 2.73-3.23), followed by baseline socioeconomic (HR, 2.63; 95% CI, 2.45-2.82) and baseline health (HR, 2.09; 95% CI, 1.95-2.23). Climate-related domains were also significantly associated, including climate socioeconomic (HR, 1.34; 95% CI, 1.21-1.48), climate health (HR, 1.31; 95% CI, 1.09-1.59), and climate extreme (HR, 1.22; 95% CI, 1.09-1.36). Baseline environment showed a modest but significant association (HR, 1.12; 95% CI, 1.03-1.21; *P* = .01).

### Interactions Between CVI Domains

As shown in eTable 4 in Supplement 1, several CVI domain pairs exhibited significant interactions for T2D risk. Baseline health positively interacted with baseline infrastructure, baseline environment, and climate health, but showed negative interactions with baseline socioeconomic, climate socioeconomic, and climate extreme domains. Baseline infrastructure demonstrated the broadest pattern of positive interactions across domains, whereas climate socioeconomic showed negative interactions with both baseline health and baseline socioeconomic.

## Discussion

In this cohort study of more than 1 million T2D-free individuals from a large, integrated US health care system in a climate-vulnerable region, we demonstrate a significant association between CVI and incident T2D. Individuals in the highest CVI quartile had a 23% higher risk of developing diabetes (HR, 1.23; 95% CI, 1.11-1.36) compared with those in the lowest quartile, even after adjusting for demographics, insurance status, cardiovascular risk factors, and baseline HbA_1c_.

This is, to our knowledge, the first study to evaluate climate vulnerability, measured using a holistic, multidimensional index, in relation to incident T2D in a large, racially and socioeconomically diverse US population. These findings expand on prior research linking social, economic, and environmental stressors to diabetes risk and align with the concept of the global syndemic, describing the interconnected epidemics of obesity, malnutrition, and climate change associated with shared structural and societal determinants.^17^ They further suggest that climate-related and societal stressors (rising temperatures, pollution, and infrastructure inequities) may indirectly contribute to metabolic dysfunction through behavioral and physiologic pathways.^6,17^

Prior studies have implicated environmental and SDOH—including air pollution,^9,10^ noise pollution,^11^ light pollution,^12^ exposure to metals^13^ or hazardous waste,^14^ extreme heat,^15^ and historic redlining^16^—in contributing to T2D risk.^6^ The CVI provides a unifying framework capturing these diverse exposures by integrating multiple systemic factors into a single composite measure. By incorporating both chronic social stressors and acute environmental threats, the CVI enables a comprehensive assessment of cumulative community-level vulnerability.

Many social factors are embedded in complex socioenvironmental systems that influence T2D risk.^24^ A meta-analysis of 23 studies reported increased diabetes incidence among individuals with low education (41%), low income (40%), and low occupational status (31%).^25^ Other evidence shows an inverse association between socioeconomic status and HbA_1c_ levels among those with T2D.^26,27^ Together with our findings, this highlights the powerful role of SDOH in shaping diabetes risk. Our study extends this evidence by emphasizing community-level climate and socioeconomic vulnerability, supporting the integration of SDOH metrics into T2D risk prediction to enhance equitable prevention.

Several mechanisms may underlie the observed association between higher CVI and increased T2D risk. Communities in areas with elevated CVI scores are often exposed to overlapping stressors such as socioeconomic deprivation, limited access to health care, environmental hazards (extreme heat, flooding, or air pollution), and infrastructure vulnerabilities.^5^ These adversities contribute to chronic metabolic disturbances through multiple pathways, including inflammation, stress-induced insulin resistance,^28,29^ malnutrition, and reduced physical activity,^30^ ultimately promoting obesity and lowering engagement with preventive health care services.^31,32^

The stratified HRs (eTable 3 in Supplement 1) indicate that baseline infrastructure, baseline socioeconomic, and baseline health domains conferred the highest risk for incident T2D, with consistent associations across models. Climate-related domains (climate health, climate socioeconomic, and climate extreme) were also significantly associated with T2D risk in models 2 and 3, which included the full cohort of over 1 million patients. However, these associations were attenuated in model 4, which incorporated baseline HbA_1c_ adjustment, but was restricted to a smaller subset (approximately 40 000 patients), likely reflecting residual confounding by glycemic status or reduced power and potential selection bias in the HbA_1c_-restricted cohort. These findings suggest that structural and social inequities are the most important contributors to CVI-related diabetes risk, while climate-specific domains may also play a meaningful role that warrants further investigation. This pattern aligns with evidence showing that neighborhood deprivation and inadequate infrastructure represent primordial risk factors for obesity and T2D. Climate-vulnerable neighborhoods often overlap with areas historically affected by redlining and economic disinvestment, compounding chronic disease disparities.^7,33,34,35^

Individuals in the highest CVI quartile are also more likely to experience compounded vulnerability, including food insecurity, poor diet quality, and limited access to healthy foods, all of which are well-established T2D risk factors.^36,37,38^ The CVI integrates these metrics, including food insecurity and a Modified Food Retail Index, within its composite score. Health-related infrastructure, including transportation access, may further mediate risk. Individuals in high-CVI areas may have lower insurance coverage and greater geographic distance from quality care facilities, hindering continuity in patient-clinician relationships and reducing opportunities for preventive services aimed at early detection of prediabetes.^7,31^

Our findings carry important public health and clinical implications. Although the mechanisms underlying the CVI-T2D association remain to be fully elucidated, the results highlight the potential value of integrating community-level social and environmental context into both clinical and public health decision-making. Although the largest associations were driven by structural and socioeconomic vulnerability, CVI’s multidomain framework provides a practical lens to assess cumulative place-based risk.

Health care systems could enhance risk assessment by using geospatial tools like the CVI to identify patients residing in highly vulnerable areas. Although CVI domains are not directly extractable from electronic health records, linkage through residential geocoding could enable targeted outreach, risk stratification, and tailored interventions. At the policy level, these results support investment in community resilience, infrastructure, and socioeconomic development—particularly in historically underserved neighborhoods. Community-based approaches such as mobile health services, culturally adapted diabetes prevention programs, and improved environmental monitoring may further address the needs of high-risk populations.

### Limitations

This study should be interpreted within the context of its limitations. First, as a retrospective cohort study within a single health care system in Texas, generalizability to broader US populations may be limited. Second, reliance on administrative data and *International Statistical Classification of Diseases, Tenth Revision, Clinical Modification* coding for diabetes diagnosis introduces potential misclassification bias, although such methods are widely used and validated in epidemiologic research.^39^ Third, despite adjustment for multiple socioeconomic and clinical factors, residual confounding remains possible because of limited data on individual-level socioeconomic status, health care use, diet, physical activity, and medication adherence. Fourth, although baseline HbA_1c_ adjustment in model 4 strengthened validity, unmeasured metabolic and lifestyle factors may persist as confounders. The smaller HbA_1c_ subset (39 665 participants vs 1 003 526 participants in models 1 to 3) may also introduce selection bias and reduce comparability. Fifth, as an observational study, causal inference cannot be established. Sixth, CVI was assigned on the basis of each patient’s most recent residential address, without accounting for potential relocation during follow-up. Seventh, census tract–level CVI may not fully capture intratract heterogeneity or temporal shifts in vulnerability, as component datasets (2017 to 2019) varied slightly in coverage.^18^ Future studies should assess interventions to mitigate diabetes risk in climate-vulnerable settings.

## Conclusions

This study found an association between the CVI and incident T2D, independently of demographic, socioeconomic, and clinical factors. The largest associations were observed in the baseline infrastructure and socioeconomic domains, highlighting the impact of community-level vulnerabilities on diabetes risk. Disparities were most evident among younger individuals, those with normal BMI, and Hispanic populations, suggesting that structural and place-based inequities may contribute to early-onset metabolic risk. These findings emphasize the need for integrated strategies that incorporate climate and SDOH into T2D prevention and management. Future research should validate these associations across diverse regions and evaluate policies that enhance community resilience, expand preventive care access, and reduce environmental and social inequities driving diabetes risk.

## Notes

Supplement 1. eAppendix. Clinical Variable Definitions eTable 1. Incidence Rates of Diabetes Across CVI Quartiles eTable 2. Incidence Rates of Type 2 Diabetes Across CVI Quartiles by Age, Race and Sex eTable 3. Hazard Ratios for Individual Climate Vulnerability Index (CVI) Domains and Incident T2DM eTable 4. Summary of Significant Interactions Between CVI Domains for Incident T2DM eFigure 1. Consort Flow Diagram eFigure 2. Maps of Climate Vulnerability Index and Cohort Distribution eFigure 3. Cox Proportional Hazard Models for CVI Quartiles & Diabetes eFigure 4. Incident Diabetes per 100 Person-Years by HbA1c Quartiles & CVI Quartiles

Supplement 2. Data Sharing Statement

## References

[1] O’Hearn M, Lara-Castor L, Cudhea F, ; Global Dietary Database. Incident type 2 diabetes attributable to suboptimal diet in 184 countries. Nat Med. 2023;29(4):982-995. doi:10.1038/s41591-023-02278-8 37069363 PMC10115653

[2] Magkos F, Hjorth MF, Astrup A. Diet and exercise in the prevention and treatment of type 2 diabetes mellitus. Nat Rev Endocrinol. 2020;16(10):545-555. doi:10.1038/s41574-020-0381-5 32690918

[3] Black JL, Macinko J. Neighborhoods and obesity. Nutr Rev. 2008;66(1):2-20. doi:10.1111/j.1753-4887.2007.00001.x 18254880

[4] Diez Roux AV, Mair C. Neighborhoods and health. Ann N Y Acad Sci. 2010;1186:125-145. doi:10.1111/j.1749-6632.2009.05333.x 20201871

[5] Rajagopalan S, Ramaswami A, Bhatnagar A, ; American Heart Association Council on Hypertension; Council on Lifestyle and Cardiometabolic Health; Council on Peripheral Vascular Disease; Council on Lifelong Congenital Heart Disease and Heart Health in the Young; Council on Cardiovascular Surgery and Anesthesia; and the American Heart Association Advocacy Coordinating Committee. Toward heart-healthy and sustainable cities: a policy statement from the American Heart Association. Circulation. 2024;149(15):e1067-e1089. doi:10.1161/CIR.0000000000001217 38436070 PMC12160618

[6] Rajagopalan S, Vergara-Martel A, Zhong J, . The urban environment and cardiometabolic health. Circulation. 2024;149(16):1298-1314. doi:10.1161/CIRCULATIONAHA.123.067461 38620080 PMC11093754

[7] Hill-Briggs F, Adler NE, Berkowitz SA, . Social determinants of health and diabetes: a scientific review. Diabetes Care. 2020;44(1):258-279. doi:10.2337/dci20-0053 33139407 PMC7783927

[8] Rajagopalan S, Brook RD, Salerno PRVO, . Air pollution exposure and cardiometabolic risk. Lancet Diabetes Endocrinol. 2024;12(3):196-208. doi:10.1016/S2213-8587(23)00361-3 38310921 PMC11264310

[9] Andersen ZJ, Raaschou-Nielsen O, Ketzel M, . Diabetes incidence and long-term exposure to air pollution: a cohort study. Diabetes Care. 2012;35(1):92-98. doi:10.2337/dc11-1155 22074722 PMC3241311

[10] Balti EV, Echouffo-Tcheugui JB, Yako YY, Kengne AP. Air pollution and risk of type 2 diabetes mellitus: a systematic review and meta-analysis. Diabetes Res Clin Pract. 2014;106(2):161-172. doi:10.1016/j.diabres.2014.08.010 25262110

[11] Eze IC, Foraster M, Schaffner E, . Long-term exposure to transportation noise and air pollution in relation to incident diabetes in the SAPALDIA study. Int J Epidemiol. 2017;46(4):1115-1125. doi:10.1093/ije/dyx020 28338949 PMC5837207

[12] Wu Y, Jiao Y, Shen P, . Outdoor light at night, air pollution and risk of incident type 2 diabetes. Environ Res. 2024;263(pt 1):120055. doi:10.1016/j.envres.2024.120055 39322059

[13] Liu B, Feng W, Wang J, . Association of urinary metals levels with type 2 diabetes risk in coke oven workers. Environ Pollut. 2016;210:1-8. doi:10.1016/j.envpol.2015.11.046 26689646

[14] Kouznetsova M, Huang X, Ma J, Lessner L, Carpenter DO. Increased rate of hospitalization for diabetes and residential proximity of hazardous waste sites. Environ Health Perspect. 2007;115(1):75-79. doi:10.1289/ehp.9223 17366823 PMC1797837

[15] Gao D, Friedman S, Hosler A, Sheridan S, Zhang W, Lin S. Association between extreme ambient heat exposure and diabetes-related hospital admissions and emergency department visits: a systematic review. Hyg Environ Health Adv. 2022;4:100031. doi:10.1016/j.heha.2022.100031 36777310 PMC9914517

[16] Motairek I, Lee EK, Janus S, . Historical neighborhood redlining and contemporary cardiometabolic risk. J Am Coll Cardiol. 2022;80(2):171-175. doi:10.1016/j.jacc.2022.05.010 35798451 PMC10411483

[17] Swinburn BA, Kraak VI, Allender S, . The global syndemic of obesity, undernutrition, and climate change: the Lancet Commission report. Lancet. 2019;393(10173):791-846. doi:10.1016/S0140-6736(18)32822-8 30700377

[18] Tee Lewis PG, Chiu WA, Nasser E, . Characterizing vulnerabilities to climate change across the United States. Environ Int. 2023;172:107772. doi:10.1016/j.envint.2023.107772 36731185 PMC10214772

[19] Environmental Defense Fund. The U.S. Climate Vulnerability Index. 2025. Accessed November 3, 2025. https://climatevulnerabilityindex.org

[20] Vieira de Oliveira Salerno PR, Lewis PGT, Chen Z, . Climate vulnerability and cardiovascular-kidney-metabolic disease in the United States. J Am Heart Assoc. 2025;14(11):e038251. doi:10.1161/JAHA.124.038251 40417800 PMC12229176

[21] Ebi KL, Capon A, Berry P, . Hot weather and heat extremes: health risks. Lancet. 2021;398(10301):698-708. doi:10.1016/S0140-6736(21)01208-3 34419205

[22] Zhang W, Villarini G, Vecchi GA, Smith JA. Urbanization exacerbated the rainfall and flooding caused by Hurricane Harvey in Houston. Nature. 2018;563(7731):384-388. doi:10.1038/s41586-018-0676-z 30429551

[23] Nasir K, Gullapelli R, Nicolas JC, . Houston Methodist cardiovascular learning health system (CVD-LHS) registry: methods for development and implementation of an automated electronic medical record-based registry using an informatics framework approach. Am J Prev Cardiol. 2024;18:100678. doi:10.1016/j.ajpc.2024.100678 38756692 PMC11096937

[24] Ramaswami A, Weible C, Main D, . A social ecological infrastructural systems framework for interdisciplinary study of sustainable city systems: an integrative curriculum across seven major disciplines. J Ind Ecol. 2012;16(6):801-813. doi:10.1111/j.1530-9290.2012.00566.x

[25] Agardh E, Allebeck P, Hallqvist J, Moradi T, Sidorchuk A. Type 2 diabetes incidence and socio-economic position: a systematic review and meta-analysis. Int J Epidemiol. 2011;40(3):804-818. doi:10.1093/ije/dyr029 21335614

[26] Bijlsma-Rutte A, Rutters F, Elders PJM, Bot SDM, Nijpels G. Socio-economic status and HbA_1c_ in type 2 diabetes: a systematic review and meta-analysis. Diabetes Metab Res Rev. 2018;34(6):e3008. doi:10.1002/dmrr.3008 29633475

[27] Bush KJ, Papacosta AO, Lennon LT, . Influence of neighborhood-level socioeconomic deprivation and individual socioeconomic position on risk of developing type 2 diabetes in older men: a longitudinal analysis in the British Regional Heart Study cohort. BMJ Open Diabetes Res Care. 2023;11(5):e003559. doi:10.1136/bmjdrc-2023-003559 37907278 PMC10619023

[28] Sørensen M, Poulsen AH, Hvidtfeldt UA, . Effects of sociodemographic characteristics, comorbidity, and coexposures on the association between air pollution and type 2 diabetes: a nationwide cohort study. Environ Health Perspect. 2023;131(2):27008. doi:10.1289/EHP11347 36802347 PMC9942819

[29] Rajagopalan S, Brook RD. Air pollution and type 2 diabetes: mechanistic insights. Diabetes. 2012;61(12):3037-3045. doi:10.2337/db12-0190 23172950 PMC3501850

[30] Mujahid MS, Maddali SR, Gao X, Oo KH, Benjamin LA, Lewis TT. The impact of neighborhoods on diabetes risk and outcomes: centering health equity. Diabetes Care. 2023;46(9):1609-1618. doi:10.2337/dci23-0003 37354326 PMC10465989

[31] Lu H, Holt JB, Cheng YJ, Zhang X, Onufrak S, Croft JB. Population-based geographic access to endocrinologists in the United States, 2012. BMC Health Serv Res. 2015;15:541. doi:10.1186/s12913-015-1185-5 26644021 PMC4672571

[32] Lòpez-DeFede A, Stewart JE. Diagnosed diabetes prevalence and risk factor rankings, by state, 2014-2016: a ring map visualization. Prev Chronic Dis. 2019;16:E44. doi:10.5888/pcd16.180470 30974073 PMC6464038

[33] Ogungbe O, Yeh HC, Cooper LA. Living within the redlines: how structural racism and redlining shape diabetes disparities. Diabetes Care. 2024;47(6):927-929. doi:10.2337/dci24-0019 38768331

[34] Ralston R, Godziewski C, Brooks E. Reconceptualising the commercial determinants of health: bringing institutions in. BMJ Glob Health. 2023;8(11):e013698. doi:10.1136/bmjgh-2023-013698 38016709 PMC10685945

[35] Manware M, Dubrow R, Carrión D, Ma Y, Chen K. Residential and race/ethnicity disparities in heat vulnerability in the United States. Geohealth. Published online December 7, 2022. doi:10.1029/2022GH000695 PMC9744626 36518814

[36] Nelson MC, Ingram SE, Dugmore AJ, . Climate challenges, vulnerabilities, and food security. Proc Natl Acad Sci U S A. 2016;113(2):298-303. doi:10.1073/pnas.1506494113 26712017 PMC4720298

[37] Hallegatte S, Rozenberg J. Climate change through a poverty lens. Nat Clim Chang. 2017;7(4):250-256. doi:10.1038/nclimate3253

[38] Hilmers A, Hilmers DC, Dave J. Neighborhood disparities in access to healthy foods and their effects on environmental justice. Am J Public Health. 2012;102(9):1644-1654. doi:10.2105/AJPH.2012.300865 22813465 PMC3482049

[39] Chen G, Khan N, Walker R, Quan H. Validating ICD coding algorithms for diabetes mellitus from administrative data. Diabetes Res Clin Pract. 2010;89(2):189-195. doi:10.1016/j.diabres.2010.03.007 20363043

