# Supplementary material for: Climate Vulnerability Index and Incident Type 2 Diabetes in a Large Integrated Health Care System
Source: JAMA Netw Open. 2025 Dec 5;8(12):e2547119. doi: 10.1001/jamanetworkopen.2025.47119 (PMC12681038; doi:10.1001/jamanetworkopen.2025.47119)
Supplement: Supplement 1. — eAppendix. Clinical Variable Definitions eTable 1. Incidence Rates of Diabetes Across CVI Quartiles eTable 2. Incidence Rates of Type 2 Diabetes Across CVI Quartiles by Age, Race and Sex eTable 3. Hazard Ratios for Individual Climate Vulnerability Index (CVI) Domains and Incident T2DM eTable 4. Summary of Significant Interactions Between CVI Domains for Incident T2DM eFigure 1. Consort Flow Diagram eFigure 2. Maps of Climate Vulnerability Index and Cohort Distribution eFigure 3. Cox Proportional Hazard Models for CVI Quartiles & Diabetes eFigure 4. Incident Diabetes per 100 Person-Years by HbA1c Quartiles & CVI Quartiles [file jamanetwopen-e2547119-s001.pdf]

Supplemental Online Content

Ardakani J, Shahid I, Gullapelli R, et al. Climate vulnerability index and incident type 2 diabetes in a large integrated health care system. *JAMA Netw Open*. 2025;8(12):e2547119. doi:10.1001/jamanetworkopen.2025.47119

eAppendix. Clinical Variable Definitions

eTable 1. Incidence Rates of Diabetes Across CVI Quartiles

eTable 2. Incidence Rates of Type 2 Diabetes Across CVI Quartiles by Age,

Race and Sex

eTable 3. Hazard Ratios for Individual Climate Vulnerability Index (CVI) Domains and Incident T2DM

eTable 4. Summary of Significant Interactions Between CVI Domains for Incident T2DM

eFigure 1. Consort Flow Diagram

eFigure 2. Maps of Climate Vulnerability Index and Cohort Distribution

eFigure 3. Cox Proportional Hazard Models for CVI Quartiles & Diabetes

eFigure 4. Incident Diabetes per 100 Person-Years by HbA1c Quartiles & CVI Quartiles

This supplemental material has been provided by the authors to give readers additional information about their work.

© 2025 Ardakani J et al. *JAMA Network Open*.

eAppendix. Clinical Variable Definitions

a. Demographics and Labs

Sex, race/ethnicity, body mass index (BMI), insurance status, hemoglobin A1c (HbA1c), low-density lipoprotein ch olesterol (LDL-C), high-density lipoprotein cholesterol (HDL-C), and triglycerides were extracted from the electroni c medical record (EMR) at the time of the first outpatient encounter.

b. Hypertension

Hypertension was defined using ICD-10-CM codes, including:

- I10 – Essential (primary) hypertension
- I11.0 – Hypertensive heart disease with heart failure
- I11.9 – Hypertensive heart disease without heart failure
- I12.0 – Hypertensive CKD with stage 5 CKD or ESRD
- I12.9 – Hypertensive CKD with stage 1–4 CKD or unspecified CKD
- I13.0 – Hypertensive heart and CKD with heart failure and stage 1–4 CKD
- I13.10 – Hypertensive heart and CKD without heart failure, with stage 1–4 CKD
- I13.11 – Hypertensive heart and CKD without heart failure, with stage 5 CKD or ESRD
- I13.2 – Hypertensive heart and CKD with heart failure and with stage 5 CKD or ESRD
- I15.0 – Renovascular hypertension
- I15.1 – Hypertension secondary to other renal disorders
- I15.2 – Hypertension secondary to endocrine disorders
- I15.8 – Other secondary hypertension
- I15.9 – Secondary hypertension, unspecified

c. Type 2 Diabetes Mellitus (T2DM)

T2DM was defined using a validated EHR-based phenotype algorithm requiring:

(1) ≥2 T2DM-related ICD-10-CM codes recorded on separate encounters at least 6 months apart,

AND

(2) At least one of the following additional criteria:

- Diabetes-specific medication use, including:

© 2025 Ardakani J et al. *JAMA Network Open*.

- Sodium-glucose cotransporter-2 (SGLT2) inhibitors
- Glucagon-like peptide-1 receptor agonists (GLP-1 RAs)
- Insulin
- Biguanides
- Sulfonamides
- Alpha-glucosidase inhibitors
- Thiazolidinediones
- Dipeptidyl peptidase-4 (DPP-4) inhibitors
- Sulfonylureas
- Aldose reductase inhibitors
- Combination agents

Abnormal glycemic laboratory criteria, including:

- Random glucose ≥200 mg/dL
- Fasting glucose >125 mg/dL
- HbA1c ≥6.5%

T2DM-specific ICD-10-CM codes included:

E11.00, E11.01, E11.10, E11.11, E11.21, E11.22, E11.29, E11.311, E11.319,

E11.3211–E11.3219, E11.3291–E11.3299, E11.3311–E11.3319,

E11.3391–E11.3399, E11.3411–E11.3419, E11.3491–E11.3499,

E11.3511–E11.3519, E11.3521–E11.3529, E11.3531–E11.3539,

E11.3541–E11.3549, E11.3551–E11.3559, E11.3591–E11.3599,

E11.36, E11.37X1–E11.37X9, E11.39, E11.40–E11.49,

E11.51, E11.52, E11.59, E11.610, E11.618,

E11.620, E11.621, E11.622, E11.628, E11.630,

E11.638, E11.641, E11.649, E11.65, E11.69, E11.8, E11.9

Excluded codes (not considered as T2DM):

- E08.x: Diabetes mellitus due to underlying condition
- E09.x: Drug- or chemical-induced diabetes mellitus

© 2025 Ardakani J et al. *JAMA Network Open*.

- E10.x: Type 1 diabetes mellitus
- E13.x: Other specified diabetes mellitus (including postprocedural or secondary)
- O24.4x: Gestational diabetes mellitus
- P70.2: Neonatal diabetes mellitus

d. Dyslipidemia

Defined as LDL cholesterol >130 mg/dL, triglycerides >150 mg/dL, or current use of lipid-lowering therapy, including:

- PCSK9 inhibitors
- Ezetimibe
- Fibrates
- Bempedoic acid
- Inclisiran
- Omega-3 fatty acids
- Bile acid sequestrants

e. BMI

Obese BMI: Defined as a BMI ≥30 kg/m²

Overweight BMI: Defined as a BMI 25-29.9 kg/m²

Normal BMI: Defined as a BMI 18.5-24.9 kg/m²

© 2025 Ardakani J et al. *JAMA Network Open*.

eTable 1: Incidence Rates of Diabetes Across CVI Quartiles

CVI Quartile (n)	Events	Person Years	IR per 100PY (95% CI)
Q1 (250882)	8,616	584,031	1.48 (1.44 - 1.51)
Q2 (250882)	8,616	567,313	1.52 (1.49 - 1.55)
Q3 (250881)	10,154	499,631	2.03 (1.99 - 2.07)
Q4 (250881)	12,766	479,210	2.66 (2.62 - 2.71)
All Quartiles	40,152	2,130,185	1.88 (1.87 - 1.90)

© 2025 Ardakani J et al. *JAMA Network Open*.

eTable 2: Incidence Rates of Type 2 Diabetes Across CVI Quartiles by Age, Race and Sex

Category	Subgroup	All Quartiles	CVI Q1	CVI Q2	CVI Q3	CVI Q4
		IR per 100 PYs (95% CI)	IR per 100 PYs (95% CI)	IR per 100 PYs (95% CI)	IR per 100 PYs (95% CI)	IR per 100 PYs (95% CI)
Age	<50	1.03 (1.01–1.05)	0.75 (0.71–0.78)	0.79 (0.76–0.83)	1.16 (1.11–1.21)	1.53 (1.47–1.59)
	≥50	2.43 (2.41–2.46)	1.91 (1.87–1.96)	2.02 (1.97–2.07)	2.61 (2.55–2.67)	3.35 (3.28–3.41)
Race	African American/Black	2.58 (2.45–2.72)	2.22 (2.06–2.38)	2.40 (2.23–2.57)	2.69 (2.51–2.88)	3.23 (3.04–3.43)
	Asian	1.98 (1.82–2.16)	1.97 (1.78–2.18)	1.78 (1.61–1.96)	1.76 (1.59–1.95)	2.80 (2.56–3.06)
	Hispanic/Latinx	2.34 (2.20–2.49)	1.73 (1.62–1.85)	1.91 (1.80–2.03)	2.49 (2.35–2.64)	3.38 (3.20–3.57)
	White	1.51 (1.47–1.56)	1.27 (1.22–1.32)	1.32 (1.26–1.38)	1.76 (1.69–1.83)	2.19 (2.12–2.27)
	Other	1.49 (1.33–1.66)	1.29 (1.13–1.47)	1.26 (1.10–1.44)	1.65 (1.47–1.86)	2.38 (2.15–2.62)
Sex	Female	1.50 (1.42–1.58)	1.17 (1.09–1.25)	1.23 (1.15–1.31)	1.67 (1.57–1.77)	2.24 (2.12–2.36)
	Male	2.36 (2.26–2.47)	1.98 (1.87–2.09)	2.04 (1.93–2.15)	2.71 (2.58–2.84)	3.46 (3.30–3.63)

© 2025 Ardakani J et al. *JAMA Network Open*.

eTable 3: Hazard Ratios for Individual Climate Vulnerability Index (CVI) Domains and Incident T2DM

CVI Domain	Model 1 HR (95% CI)	Model 1 P val	Model 2 HR (95% CI)	Model 2 P val	Model 3 HR (95% CI)	Model 3 P val	Model 4 HR (95% CI)	Model 4 P val
Baseline Health	4.72 (4.42 - 5.04)	<.001	3.77 (3.53 - 4.03)	<.001	2.09 (1.95 - 2.23)	<.001	1.87 (1.48 - 2.36)	<.001
Baseline Socioeconomic	6.24 (5.82 - 6.68)	<.001	5.08 (4.74 - 5.45	<.001	2.63 (2.45 - 2.82)	<.001	1.97 (1.53 - 2.54)	<.001
Baseline Infrastructure	8.23 (7.57 - 8.95)	<.001	6.76 (6.22 - 7.35)	<.001	2.97 (2.73 - 3.23)	<.001	2.29 (1.7 - 3.08)	<.001
Baseline Environment	0.98 (0.9 - 1.06)	.57	0.95 (0.88 - 1.02)	.1771	1.12 (1.03 - 1.21)	.01	0.99 (0.75 - 1.33)	.97
Climate Health	1.00 (0.83 - 1.21)	.96	1.47 (1.22 - 1.78)	<.001	1.31 (1.09 - 1.59)	.01	1.07 (0.54 - 2.12)	.85
Climate Socioeconomic	1.97 (1.78 - 2.18)	<.001	2.15 (1.94 - 2.39)	<.001	1.34 (1.21 - 1.48)	<.001	1.18 (0.79 - 1.76)	.43
Climate Extreme	1.93 (1.73 - 2.16)	<.001	2.04 (1.82 - 2.28)	<.001	1.22 (1.09 - 1.36)	<.001	1.30 (0.86 - 1.98)	.22

Model 1: Unadjusted (n=1,003,526)
Model 2: Adjusted for age, sex, race/ethnicity (n=1,003,526)
Model 3: Adjusted for model 2 + insurance status and cardiovascular risk factors (hypertension, obesity, dyslipidemia, and smoking) (n=1,003,526)

Model 4: Adjusted for Model 3 + HbA1c (n=39,665).

© 2025 Ardakani J et al. *JAMA Network Open*.

eTable 4: Summary of Significant Interactions Between CVI Domains for Incident T2DM

	Baseline Health	Baseline Socioeconomic	Baseline Infrastructure	Baseline Environment	Climate Health	Climate Socioeconomic	Climate Extreme
Baseline Health		–	+	+	+	–	–
Baseline Socioeconomic			–	+	+	–	–
Baseline Infrastructure				+	+	±	–
Baseline Environment					–	+	+
Climate Health						±	+
Climate Socioeconomic							±

+ indicates statistically significant positive interaction (HR > 1, P < .05); − indicates statistically significant negative interaction (HR < 1, P < .05); ± indicates not statistically significant (P ≥ .05). Interactions were modeled using unadjusted Cox proportional hazards models.

© 2025 Ardakani J et al. *JAMA Network Open*.

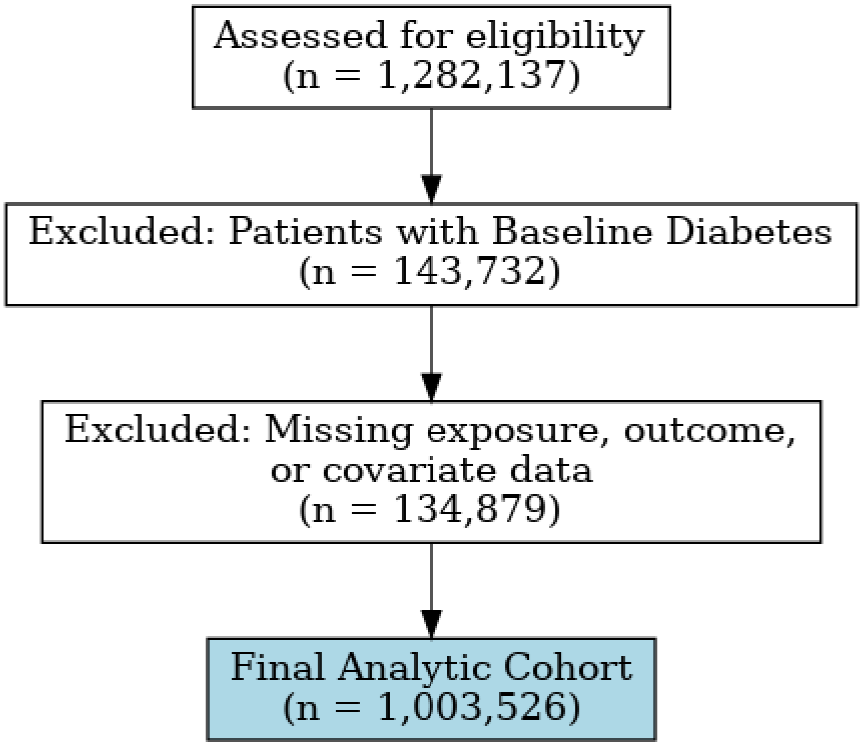

eFigure 1. Consort Flow Diagram

© 2025 Ardakani J et al. *JAMA Network Open*.

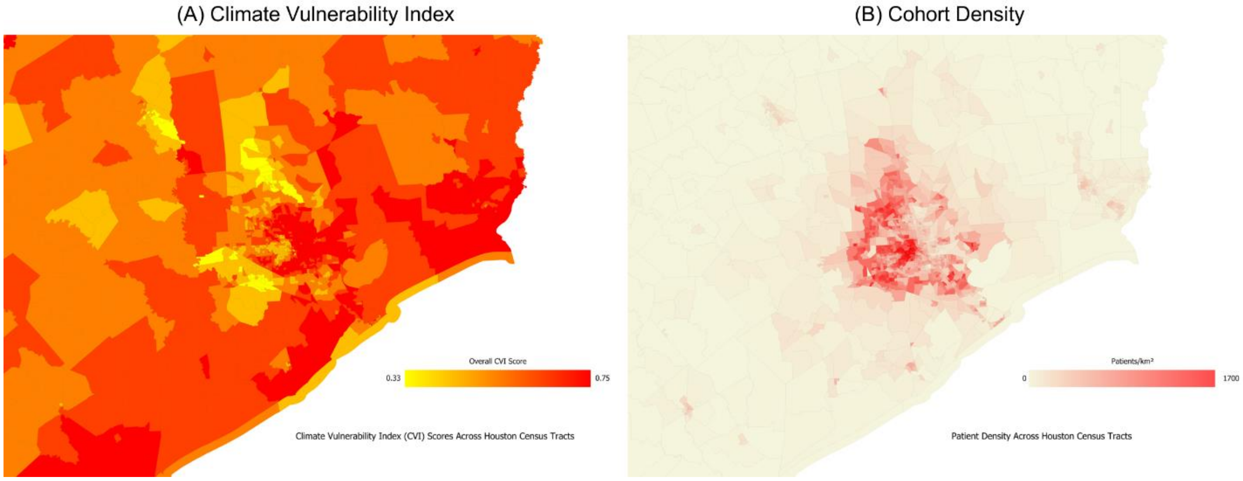

eFigure 2. Maps of Climate Vulnerability Index and Cohort Distribution

© 2025 Ardakani J et al. *JAMA Network Open*.

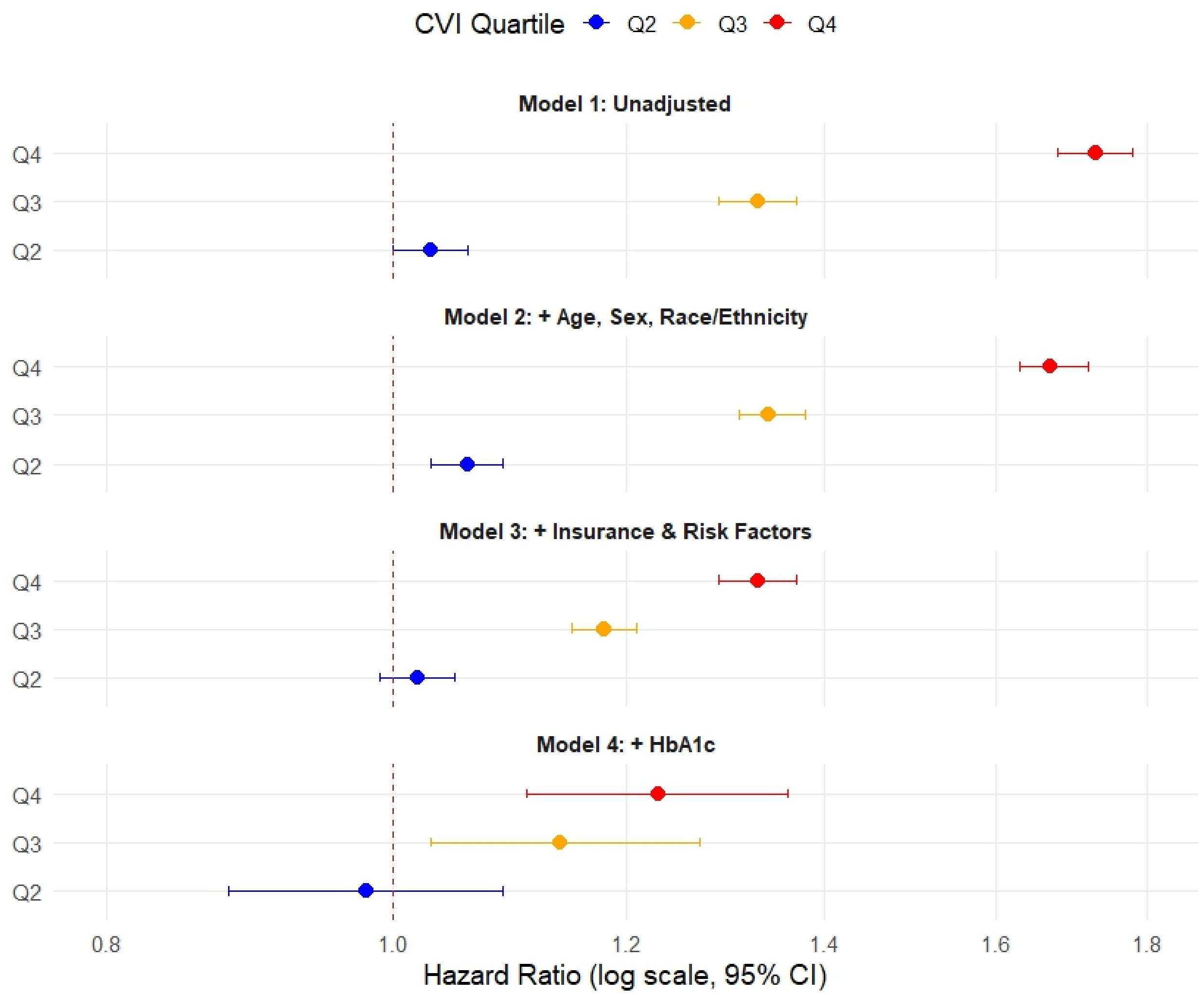

eFigure 3. Cox Proportional Hazard Models for CVI Quartiles & Diabetes

© 2025 Ardakani J et al. *JAMA Network Open*.

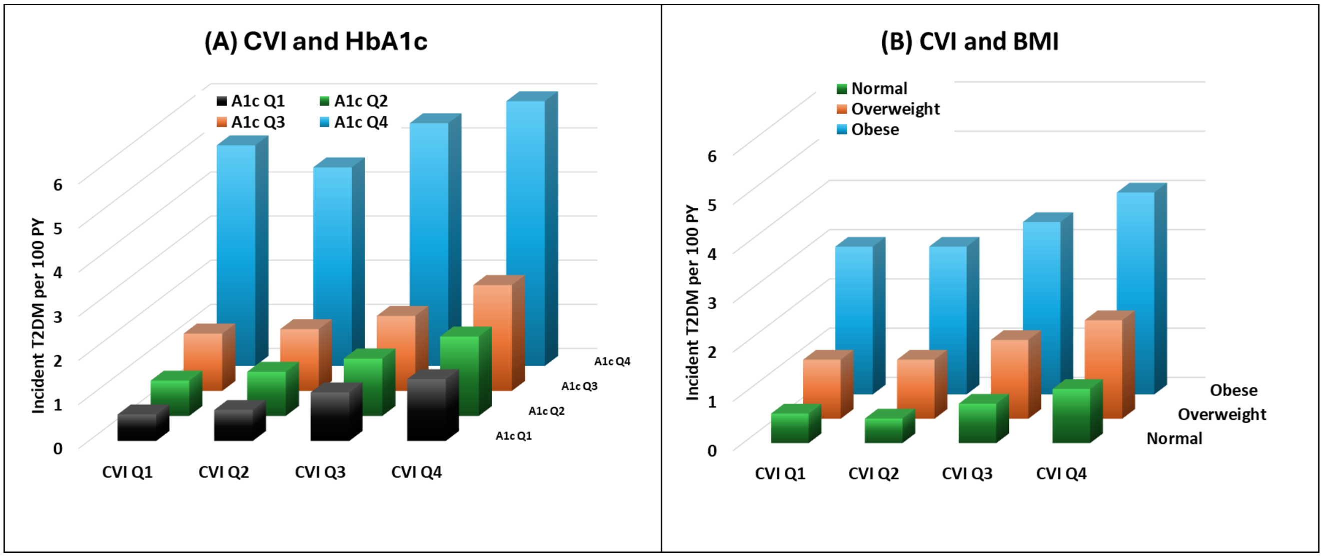

eFigure 4: Incident Diabetes per 100 Person-Years by HbA1c Quartiles & CVI Quartiles

© 2025 Ardakani J et al. *JAMA Network Open*.
