# Supplementary material for: Climate Vulnerability Index and Incident Type 2 Diabetes in a Large Integrated Health Care System
Source: JAMA Netw Open. 2025 Dec 5;8(12):e2547119. doi: 10.1001/jamanetworkopen.2025.47119 (PMC12681038; doi:10.1001/jamanetworkopen.2025.47119)
Supplement: Supplement 2. — Data Sharing Statement [file jamanetwopen-e2547119-s002.pdf]

Data Sharing Statement

Ardakani. Climate Vulnerability Index and Incident Type 2 Diabetes in a Large Integrated Health Care System. *JAMA Netw Open*. Published December 05, 2025. doi:10.1001/jamanetworkopen.2025.47119

Data
Data available: No

Additional Information
Explanation for why data not available: The data used in this study are derived from the Houston Methodist CVD Learning Health System Registry and include protected health information. Due to institutional policies, HIPAA regulations, and IRB requirements, these data cannot be shared publicly to protect patient privacy and confidentiality.
